# ImmunoPET imaging of human CD8^+^ T cells with novel ^68^Ga-labeled nanobody companion diagnostic agents

**DOI:** 10.1186/s12951-021-00785-9

**Published:** 2021-02-09

**Authors:** Haitao Zhao, Chao Wang, Yanling Yang, Yan Sun, Weijun Wei, Cheng Wang, Liangrong Wan, Cheng Zhu, Lianghua Li, Gang Huang, Jianjun Liu

**Affiliations:** 1 Department of Nuclear Medicine, Institute of Clinical Nuclear Medicine, Renji Hospital, School of Medicine, Shanghai Jiao Tong University, 1630 Dongfang Rd, Shanghai, 200127 China; 2 SmartNuclide Biopharma Co. Ltd, 218 Xinghu St., BioBAY A4-202, Suzhou Industrial Park, Suzhou, China; 3 School of Pharmacy, Yantai University, No. 32 Road QingQuan, Laishan District, Yantai, 264005 China; 4 State Key Laboratory of Oncogenes and Related Genes, Shanghai Cancer Institute, 1630 Dongfang Rd, Shanghai, 200127 China; 5 Shanghai Key Laboratory of Molecular Imaging, Shanghai University of Medicine and Health Sciences, Shanghai, 201318 China

**Keywords:** CD8^+^ T lymphocytes, ImmunoPET, Nanobody, Immunotherapy, Companion diagnostics

## Abstract

**Background:**

Although immunotherapy has revolutionized treatment strategies for some types of cancers, most patients failed to respond or obtain long-term benefit. Tumor-infiltrating CD8^+^ T lymphocytes are closely related to the treatment outcome and prognosis of patients. Therefore, noninvasive elucidation of both systemic and tumor-infiltrating CD8^+^ T lymphocytes is of extraordinary significance for patients during cancer immunotherapy. Herein, a panel of ^68^Ga-labeled Nanobodies were designed and investigated to track human CD8^+^ T cells in vivo through immuno-positron emission tomography (immunoPET).

**Results:**

Among the screened Nanobodies, SNA006a showed the highest binding affinity and specificity to both human CD8 protein and CD8^+^ cells in vitro, with the equilibrium dissociation constant (*K*_*D*_) of 6.4 × 10^−10^ M and 4.6 × 10^−10^ M, respectively. ^68^Ga-NOTA-SNA006 was obtained with high radiochemical yield and purity, and stayed stable for at least 1 h both in vitro and in vivo. Biodistribution and Micro-PET/CT imaging studies revealed that all tracers specifically concentrated in the CD8^+^ tumors with low accumulation in CD8^−^ tumors and normal organs except the kidneys, where the tracer was excreted and reabsorbed. Notably, the high uptake of ^68^Ga-NOTA-SNA006a in CD8^+^ tumors was rapid and persistent, which reached 24.41 ± 1.00% ID/g at 1.5 h after intravenous injection, resulting in excellent target-to-background ratios (TBRs). More specifically, the tumor-to-muscle, tumor-to-liver, and CD8^+^ to CD8^−^ tumor was 28.10 ± 3.68, 5.26 ± 0.86, and 19.58 ± 2.70 at 1.5 h, respectively. Furthermore, in the humanized PBMC-NSG and HSC-NPG mouse models, ^68^Ga-NOTA-SNA006a accumulated in both CD8^+^ tumors and specific tissues such as liver, spleen and lung where human CD8 antigen was overexpressed or CD8^+^ T cells located during immunoPET imaging.

**Conclusions:**

^68^Ga-NOTA-SNA006a, a novel Nanobody tracer targeting human CD8 antigen, was developed with high radiochemical purity and high affinity. Compared with other candidates, the long retention time, low background, excellent TBRs of ^68^Ga-NOTA-SNA006a make it precisely track the human CD8^+^ T cells in mice models, showing great potential for immunotherapy monitoring and efficacy evaluation.

## Background

Cancer immunotherapy mainly imply treatments with immune checkpoint inhibitors, adoptive cell therapy (chimeric antigen receptor T cells, CAR-T), and oncolytic viruses and cancer vaccines [1, 2]. The rapid development of cancer immunotherapy has led to a dramatic transformation in the treatment of multiple solid and hematologic malignancies. However, poor treatment response caused by heterogeneity and evolutionary characteristics of tumors as well as concomitant adverse reactions are impeding the wide application. Companion diagnostics (CDx) is the cornerstone of cancer precision medicine. Due to precise patient stratification and timely response assessment, CDx can improve the treatment efficacy while ensuring safety and reducing medical costs. One advanced modality of CDx is immuno-positron emission tomography (immunoPET), which delicately combines the extraordinary targeting specificity of monoclonal antibodies and the superior sensitivity of PET [3, 4]. Our previous studies have shown that immunoPET can not only visualize heterogeneous expression of tumor antigens in a non-invasive manner [5–7], but also depict the whole process of immunotherapy dynamically and quantitatively [8].

Tumor-infiltrating CD8^+^ T lymphocytes are commonly regarded as the core of cancer immunotherapy because they specifically recognize endogenous antigen peptide MHCI complex and kill tumor cells [9]. It has been reported that CD8^+^ T cells have prognostic value in multiple solid tumors, including colorectal cancer, triple-negative breast cancer, melanoma, head and neck squamous cell carcinoma, gastric cancer, lung cancer, and liver cancer [10]. Therefore, elucidating the spatial distribution and status of CD8^+^ T cells in vivo through CDx has an inestimable application prospect in personalized cancer immunotherapy. ImmunoPET, rather than traditional immunohistochemistry, provides more comprehensive and accurate information about patients, facilitating identification of patients most likely to respond to and benefit from immunotherapy. Moreover, therapeutic efficacy can be monitored and evaluated on treatment by immunoPET imaging, thus helping to rationally formulate or adjust treatment project in time to improve outcomes [11].

CD8^+^ T cells targeting agents based on antibodies, antibody fragments [12–14], diabodies [15], and minibodies [16] usually show long circulation time. Thus, isotopes of long half-lives (e.g., ^89^Zr of *t*_1/2_ = 78.4 h and ^64^Cu of *t*_1/2_ = 12.7 h) are needed for the radiolabeling [3]. As a result, tedious waiting time or multiple imaging attempts post-injection (p.i.) might be necessary to achieve desired results with satisfactory tumor-to-background ratio (TBR). In addition, immune toxicity and ionizing radiation damage might be unavoidable under certain circumstances. Nanobody, the variable domain of a heavy chain antibody (V_HH_), is the smallest antigen-binding derivative with molecular weight around 15 kDa and diameter < 4 nm. As a molecular probe, it has the advantages of high affinity, strong stability, low immunogenicity, fast clearance, and strong tissue penetration [17].

**Fig. 1:**
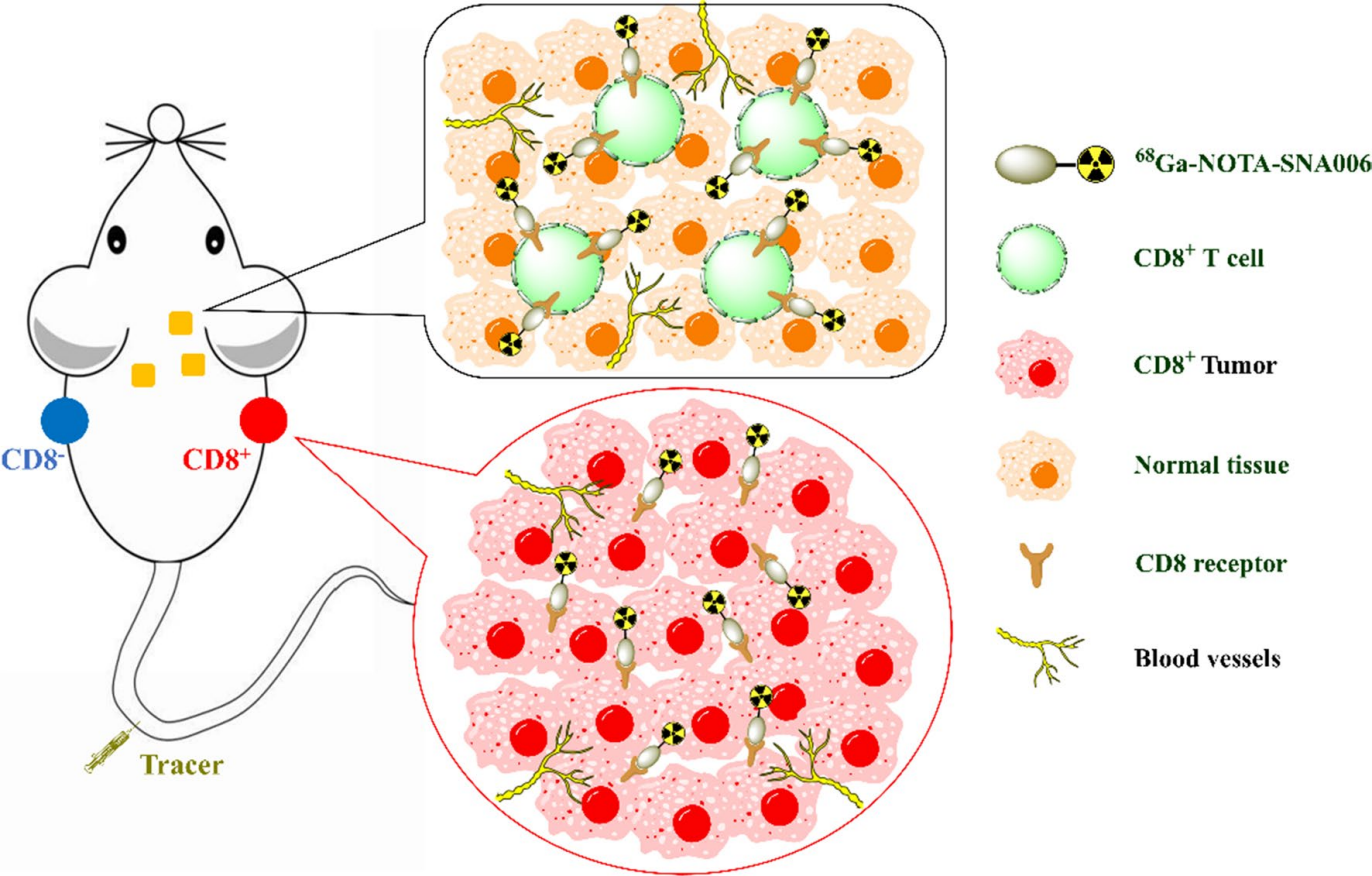
Schematic illustration of ^68^Ga-NOTA-SNA006 immunoPET in tracking CD8^+^ cells

Based on a series of newly obtained Nanobodies targeting human CD8, we constructed ^68^Ga-labeled tracers for immunoPET imaging of human CD8 antigen in the current study. Among these CDx agents, ^68^Ga-NOTA-SNA006a showed the highest targeting specificity, affinity as well as excellent pharmacokinetic characteristics. Furthermore, ^68^Ga-NOTA-SNA006a immunoPET imaging clearly visualized the distribution of CD8^+^ cells including human CD8^+^ T cells in tumors and across the whole body (Fig. 1), demonstrating great potential of the novel imaging technique for optimizing cancer immunotherapy.

## Methods

### Materials and reagents

All reagents and consumables were obtained from Sigma-Aldrich unless otherwise stated. The chelating reagent *p*-SCN-Bn-NOTA was purchased from Macrocyclics (Plano, USA). Mass spectrometry (MS) was performed with the high-resolution LTQ-Orbitrap XL mass spectrometer interfaced with a heated electrospray ionization (H-ESI) source (Thermo Scientific, San Jose, CA) and the data was analyzed with Thermo Biopharma Finder 3.0. Gallium-68 (^68^Ga, *t*_1/2_ = 1.1 h) was obtained from a ^68^Ga/^68^Ge generator (IGG-100, Eckert & Ziegler AG) and ^125^I was purchased from Atom Kexing Pharmaceuticals Company (Shanghai, China). High Performance Liquid Chromatography (HPLC) was performed on Agilent 1260 system with a SEC chromatogram (G3000SWXL, TOSOH).

### Production and purification of nanobodies

All SNA006 Nanobodies named SNA006a, SNA006c and SNA006d were provided by SmartNuclide Biopharma (Suzhou, China). Briefly, Anti-CD8α nanobody DNA fragments were cloned into pcDNA4 plasmid (Invitrogen, Cat V86220) for expression in mammalian cells with His tag fusion. Human embryonic kidney suspension-adapted cells HEK293F were cultured in suspension according to standard protocol to high density using serum-free media. Starter cultures (30–100 mL) were maintained in 250 mL conical cell culture flasks and were transiently transfected with above mentioned plasmids using a branched version of polyethylenimine (PEI), following standard protocol. After being cultured for 5–6 days after transfection, secreted nanobodies were harvested by centrifuging culture mixture at 3000×*g* for 5 min and collecting the supernatant. Intracellular proteins were harvested by centrifuging cells at 3000×*g* for 5 min. Nanobodies were purified with an Ni column, followed by ion-exchange chromatography (HiTrap S HP; GE Healthcare). The purified nanobodies were dialyzed against phosphate-buffered saline (PBS) for further use.

### Cell culture and animal models

Mouse colon cancer cell line MC38 and human CD8 transfected MC38 (hereafter referred to as MC38-CD8) cell line as well as human peripheral blood mononuclear cell (PBMC)-NSG mouse models were provided by SmartNuclide Biopharma. Cells were cultured in Dulbecco’s Modified Eagle Medium (DMEM) with 10% FBS (Gibco) at 37 °C in a humidified atmosphere containing 5% CO_2_. Female BALB/c nude mice (4–5 weeks of age) were purchased from Renji Hospital Experimental Animal Center (Shanghai, China). Haemopoietic stem cell (HSC)-NPG mouse models were provided by Vitalstar Biotechnology Co. Ltd (Beijing, China). All animal experiments were performed in accordance with the guidelines of Renji Institutional Animal Care. To establish subcutaneous tumor model, about 5 × 10^6^ of MC38 and MC38-CD8 cells in 100 μL phosphate buffer saline (PBS) were inoculated subcutaneously into the left (MC38) and right (MC38-CD8) front flanks of the mice, respectively. The animals were used for in vivo imaging studies when the tumor volume reached 200–300 mm^3^ (1–2 weeks after inoculation).

### Chemistry and radiochemistry

The purity of Nanobodies was detected by sodium dodecyl sulfate polyacrylamide gel electrophoresis (SDS-PAGE). Precursors NOTA-SNA006a, c, and d were synthesized by conjugation of *p*-SCN-Bn-NOTA with Nanobodies SNA006a, c and d according to a previous study [18]. Both Nanobodies and precursors were characterized by mass spectrometry. Enzyme-linked immunosorbent assay (ELISA) experiments were conducted to verify the biological activity of the precursors. For radiolabeling, ^68^GaCl_3_ solution (2 mL) was eluted from ^68^Ge/^68^Ga generator with 0.1 M HCl, and then sodium acetate aqueous solution (1 M, 300 μL) and NOTA-SNA006 (10 nM) precursors were added to the system. The reaction was incubated at room temperature for 10 min and purified with a PD-10 column (Cytiva) to obtain ^68^Ga-NOTA-SNA006 saline solution. The radiochemistry purity and stability of ^68^Ga-NOTA-SNA006a were measured by TLC and HPLC with 0.1 M citric acid and 200 mM NaCl as mobile phase (1 mL/min).

### Surface plasmon resonance

The affinity of Nanobodies SNA006a, c and d binding to human CD8 protein were tested by surface plasmon resonance (SPR). All measurements were performed on a Biacore T200 device (GE Healthcare) at 25 °C with Hepes-buffered saline (0.01 M HEPES pH 7.4, 0.15 M NaCl, 3 mM ethylenediaminetetraacetic acid, 0.005% Tween 20) as the running buffer. Briefly, different dilutions of the three nanobodies were run at 50 µL/min on a CM5 sensor chip with high density of human CD8 protein and specific binding signals (response units, RU) were recorded. Nanobody dilutions were allowed to bind with the target protein for 300 s and dissociation was monitored for 180 s. The equilibrium dissociation constant *K*_*D*_ was calculated by fitting the obtained sensor grams to theoretical curves using Biacore Evaluation software.

### Binding affinity assay

The cell-binding assay of Nanobodies SNA006a, c and d to MC38 and MC38-CD8 cells were analyzed. ^125^I-labeled Nanobodies were firstly prepared respectively using Iodogen (Invitrogen) and purified with a PD-10 column. One mL PBS solution was added to each tube containing fresh MC38 or MC38-CD8 cells pellet (1 × 10^6^). The cells in all tubes were re-suspended. Then, 0.5 mL/37 kBq of the ^125^I-SNA006 solution was added to each tube of two groups. After incubating at 4 °C for 4 h, 0.5 mL of the supernatant was collected after vigorous centrifugation. The radioactivity of both samples (cell pellet plus 0.5 mL medium, A, and 0.5 mL medium, B) was measured. The radioactivity was measured using a γ-counter (2470 Wizard2, Perkin Elmer, Massachusetts, USA). The percent of added dose (AD %) were expressed as AD % ((A − B)/(A + B) × 100%) per 10^6^ cells.

The saturation binding of SNA006 Nanobodies with MC38-CD8 cells divided into two groups including total binding and nonspecific binding were also performed. MC38-CD8 cells were deposited in 96-well filter plates (1 × 10^5^ cells/well), and increasing concentrations of ^125^I-SNA006 solution (0.1 ~ 100 nM/L) were added. For nonspecific combination group additional unlabelled SNA006 protein (2.5 mM/L) was added. After incubating at 4 °C for 4 h each well was washed with PBS buffer and measured using a calibrated γ counter. The results of saturation curve fitting were obtained by Prism 5.0 (GraphPad Software, Inc.) according to the single point combination model.

The competition binding assay was assessed in vitro. MC38-CD8 cells were incubated with 200,000 CPM of ^125^I-SNA006a and increasing concentrations of (10^−4^~2000 nM/L) competitive inhibitors at 4 °C for 4 h. The cells were then washed with PBS buffer and collected. The radioactivity was measured using a γ-counter. The results were processed by Prism 5.0 (GraphPad Software, Inc.), and the competition curve with the logarithm of the concentration was then fitted.

### Micro-PET/CT imaging

Mice PET/CT imaging was performed using a Micro-PET/CT system (IRIS PET/CT, Inviscan, Strasbourg, France). Briefly, female nude mouse models were injected via the tail vein with 3.7 MBq of ^68^Ga-NOTA-SNA006a, c and d respectively, and then anesthetized through inhalation of 2% isoflurane. Static imaging at 0.5, 1, 1.5 and 2 h p.i. and dynamic images for 1 h were performed. PBMC-NSG and HSC-NPG mice bearing CD8^+^ and CD8^−^ tumors as well as control (NSG and NPG) mice were injected with ^68^Ga-NOTA-SNA006a and images were obtained at 1 h p.i. Blocking groups were simultaneously injected with an overdose (about 500 μg) of unlabeled SNA006a. We also carried out ^18^F-FDG PET/CT and ^125^I-SNA006 single photon emission computed tomography (SPECT) imaging in nude mice tumor model. The data were reconstructed using Monte-Carlo based three-dimensional ordered subset expectation maximization (Monte-Carlo based 3D OSEM). Images and regions of interest (ROIs) were processed using Osirix MD software.

### Biodistribution study

Female nude mice with subcutaneous MC38 and MC38-CD8 xenografts were respectively injected with 0.37 MBq of ^68^Ga-NOTA-SNA006a, c and d respectively to evaluate the distribution of radiotracers in tumor tissues and major organs (n = 4 per group). Mice were sacrificed and dissected at 1 and 1.5 h p.i. Blood, tumor, major normal tissues and organs were collected and weighed, and the radioactivity of them was measured by a γ-counter. The results are presented as percentage of injected dose per gram of tissue (%ID/g).

### Flow cytometry and immunofluorescence staining

The expression level of CD8 antigen in CD8 transfected cells, human CD8^+^ T cells, PBMC-NSG and HSC-NPG mouse models was determined by flow cytometry as previously reported [12]. Both CD8^+^ and CD8^−^ xenografts were surgically removed and paraffin-embedded. Slices (10 μm) were obtained and stained with human CD8 alpha monoclonal antibody (Abcam, USA). All slices were visualized under a confocal microscope.

## Results

### Synthesis and characterization of ^68^Ga-NOTA-SNA006

**Fig. 2:**
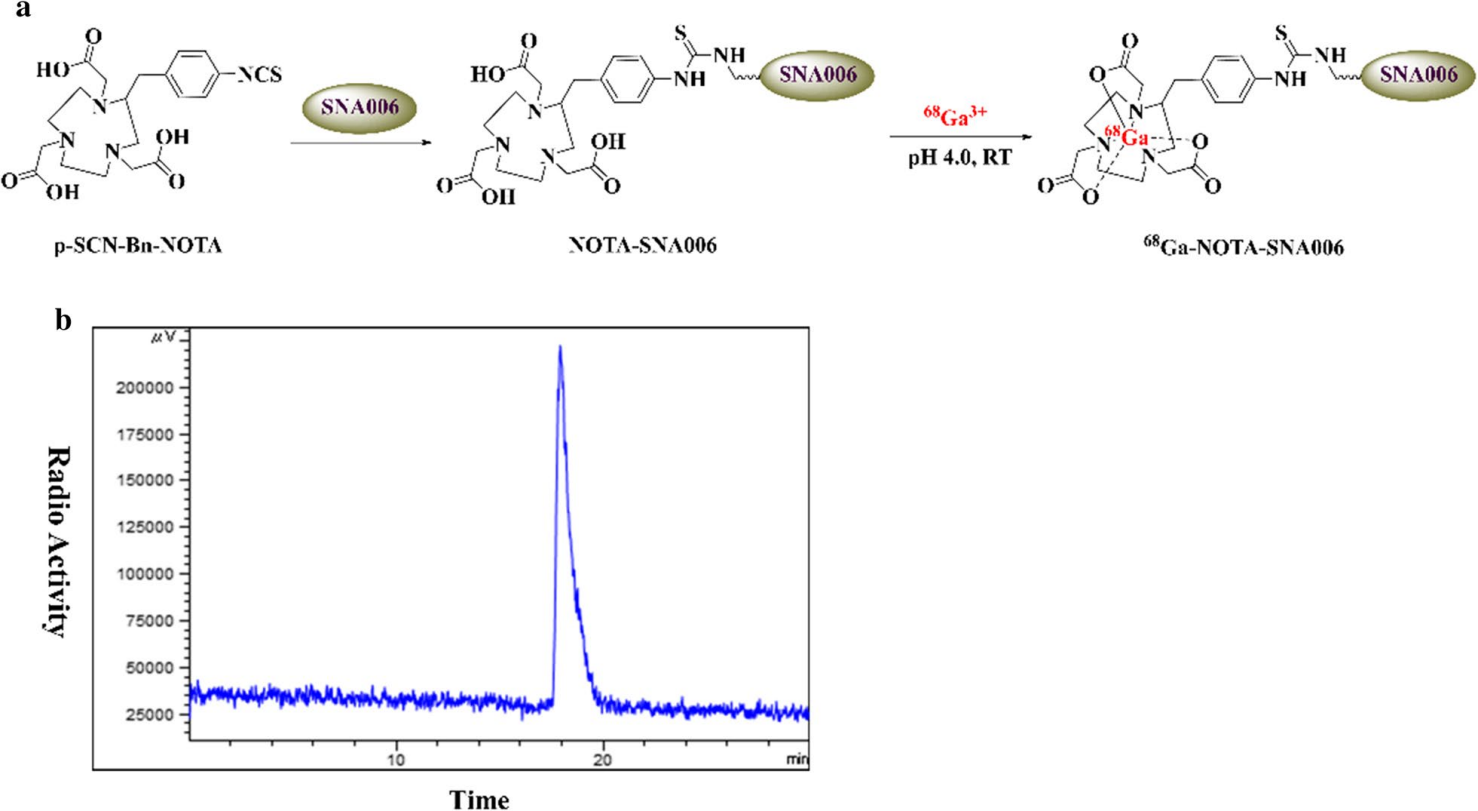
**a** Synthetic scheme of ^68^Ga-NOTA-SNA006. **b** Radio-HPLC analysis for radiochemistry purity of ^68^Ga-NOTA-SNA006

Precursors NOTA-SNA006a, c and d for radiolabel were prepared by condensation coupling of the amino groups of three different Nanobodies with the isothiocyanate group of *p*-SCN-Bn-NOTA, respectively (Fig. 2a). The molecular weight of SNA006 Nanobodies was detected by SDS-PAGE (Additional file 1: Fig. S1A) and further accurately determined by MS (Additional file 1: Fig. S1B, 15380 Da for SNA006a, 14946 Da for SNA006c and 15359 Da for SNA006d). The MS results of NOTA-SNA006 (Additional file 1: Fig. S2A) revealed that a mixture of each precursor with variable numbers of NOTA ligands was obtained because of the presence of multiple free amino groups in the Nanobody skeleton. The average number of NOTA attached to each SNA006a, c and d was calculated through MS (2.08, 1.07 and 0.60 for SNA006a,c and d, respectively). ELISA studies were also performed and similar binding curves of NOTA-SNA006 conjugates and SNA006 Nanobodies to CD8 antigen were obtained (Additional file 1: Fig. S2B), indicating that NOTA coupling does not affect the biological activity of the Nanobodies.

^68^Ga-NOTA-SNA006 was produced at room temperature via the chelation of ^68^Ga and precursors in sodium acetate buffer (pH = 4.0), and impurities were further removed with PD-10 column. TLC and HPLC profiles (Additional file 1: Fig. S3) show that, ^68^Ga-NOTA-SNA006a was prepared with radiochemical purity > 98% and remained intact for at least 1 h in vitro (phosphate buffer, fetal bovine serum) and in vivo.

### In vitro binding assays

**Fig. 3:**
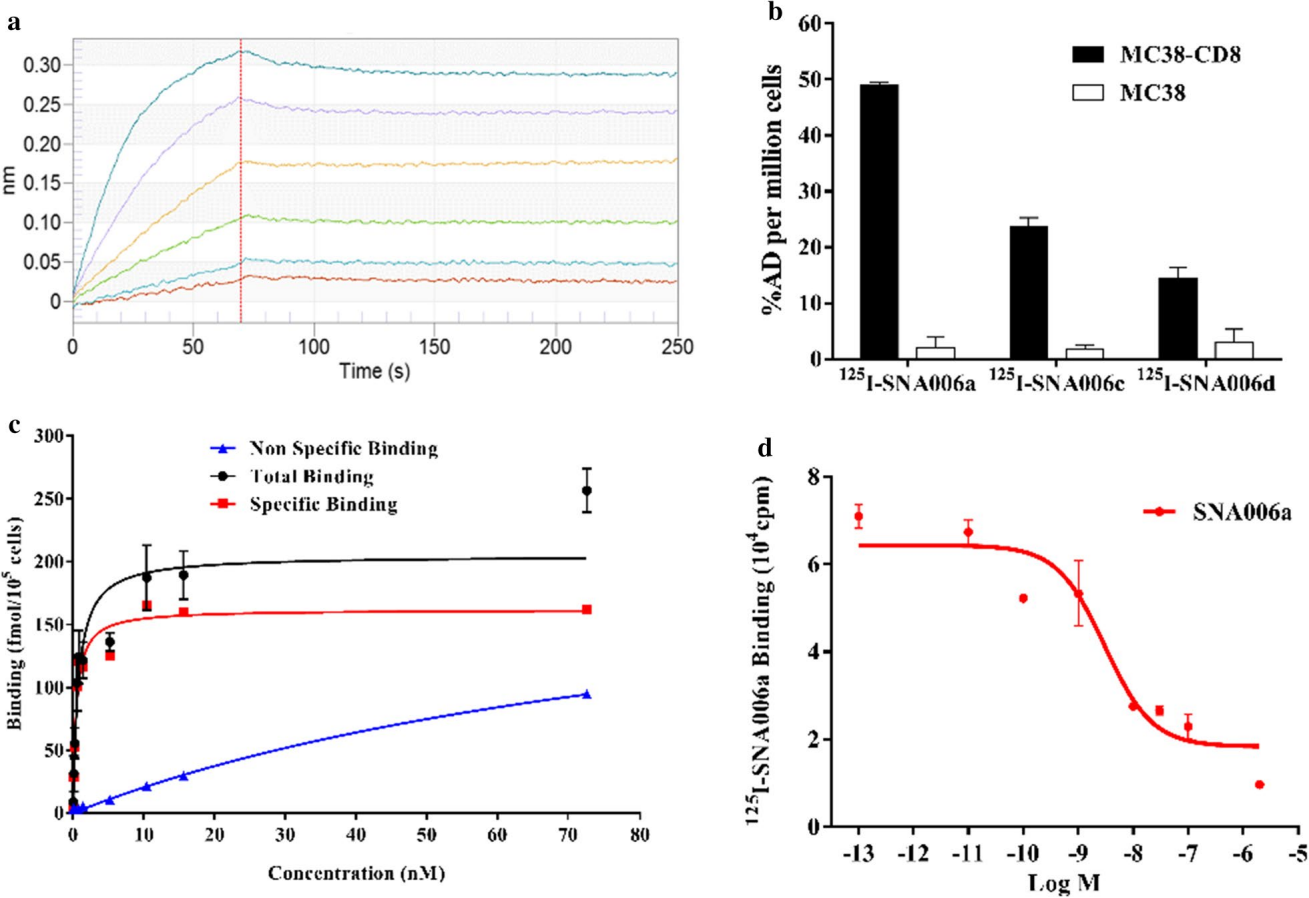
In vitro binding studies. **a** Affinity/kinetics surface plasmon resonance study of SNA006a interacting with immobilized recombinant human CD8 protein. **b** The uptake of ^125^I-SNA006 in MC38-CD8 (CD8-positive) and MC38 (CD8-negative) cells at 4 h. **c** The saturation binding curve of ^125^I-SNA006 to MC38-CD8 cells at 4 h. **d** The competition binding curve of ^125^I-SNA006 to MC38-CD8 cells at 4 h

The binding capacity of Nanobodies to recombinant human CD8 protein was determined by the SPR test (Fig. 3a, Additional file 1: Fig. S4A & 4B), and all of them showed strong binding affinity (*K*_*D*_) with high binding rate constant (*k*_*a*_) and low dissociation rate constant (*k*_*d*_), especially for SNA006a (*K*_*D*_ = 0.64 nM, *k*_*a*_ = 4.83 × 10^5^ 1/Ms and *k*_*d*_ = 3.11 × 10^−4^ 1/s). ^125^I-labeled SNA006 was prepared for in vitro evaluation of binding affinity. The results revealed that uptake of ^125^I-SNA006a, c and d at 4 h in MC38-CD8 cells (CD8-positive) were much higher than that in the parental MC38 cells (CD8-negative), suggesting the specific binding of SNA006 to cells in a CD8-dependent manner (Fig. 3b). Subsequently, the fitted binding curves confirmed the specific binding results quantitatively (Fig. 3c, Additional file 1: Fig. S4C & D). As we expected, they all demonstrated remarkable binding values to CD8^+^ cells: SNA006a (*K*_*D*_ = 0.46 nM), SNA006c (*K*_*D*_ = 3.84 nM), SNA006d (*K*_*D*_ = 1.48 nM). The above results showed that SNA006a possessed the most impressive binding affinity and specificity to CD8 antigen. Furthermore, the competitive binding capability of SNA006a to CD8-positive cells was measured and the calculated IC50 was 3.02 nM (Fig. 3d).

### Micro-PET/CT imaging

**Fig. 4:**
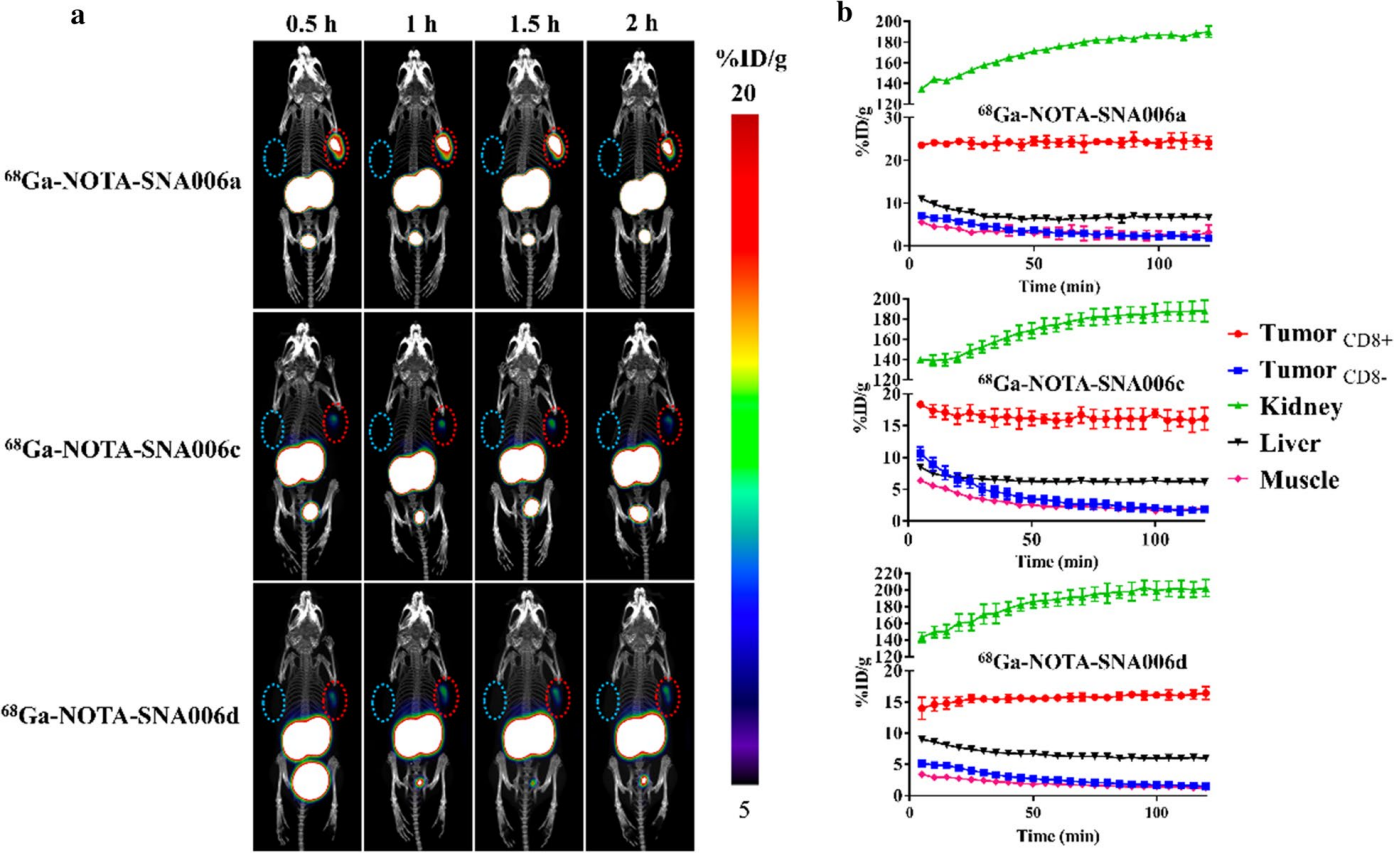
Micro-PET/CT imaging studies of tumor-bearing models (n = 3). **a** Static PET/CT scanning of both CD8^+^ MC38-CD8 (right, red circle) and CD8^−^ MC38 (blue circle) tumor-bearing nude mice models at 0.5, 1, 1.5 and 2 h p.i. of about 3.7 MBq of ^68^Ga-NOTA-SNA006. **b** The quantitative time-radioactivity curves of ^68^Ga-NOTA-SNA006 in tumors and major normal tissues analyzed according to the quantification analysis of dynamic PET/CT imaging over 0–2 h

As shown in Fig. 4a, the static Micro-PET/CT imaging of ^68^Ga-NOTA-SNA006a, c and d was performed in mice model bearing both CD8^+^ (right) and CD8^−^ (left) tumors (Additional file 1: Fig. S5A) at 0.5, 1, 1.5, and 2 h p.i. All tracers possessed high kidney and bladder retention with a fairly clean background in other normal organs and tissues. CD8^+^ tumors could be clearly visualized at all imaging time points while CD8^−^ tumors were barely visible. ^68^Ga-NOTA-SNA006a showed the optimal imaging contrast at 1 and 1.5 h p.i., which was consistent with in vitro affinity results. Blocking with cold SNA006a significantly reduced uptake of ^68^Ga-NOTA-SNA006a in the CD8^+^ tumors, with the uptake in other organs or tissues largely unchanged (Additional file 1: Fig. S5B). We also conducted ^18^F-FDG PET/CT imaging, and the two types of tumors could hardly be distinguished due to the non-specific uptake of ^18^F-FDG (Additional file 1: Fig. S5B). In addition, SPECT imaging with ^125^I-SNA006 agents at 1 h p.i. further consolidated the ^68^Ga-NOTA-SNA006 immunoPET imaging results (Additional file 1: Fig. S5C), showing prominent uptake in the CD8^+^ tumor but not in the CD^−^ tumor.

Dynamic scanning was performed for 2 h with the above mice model, and quantitative time-radioactivity curves of major organs and tissues were displayed in Fig. 4b. Similar distribution and metabolism characteristics of ^68^Ga-NOTA-SNA006a, c and d in vivo were observed. Rapid and high uptake of all the tracers in CD8^+^ tumors was observed at early time p.i., and the plateau lasted for 2 h. Renal uptake was most concentrated and increased slightly over time, while uptake in other tissues such as liver, muscle and CD8^−^ tumors was relatively low and decreased. Quantitatively, ^68^Ga-NOTA-SNA006a possessed the highest uptake in the CD8^+^ tumors and equivalent background uptake compared with its cousins.

### Biodistribution

**Fig. 5:**
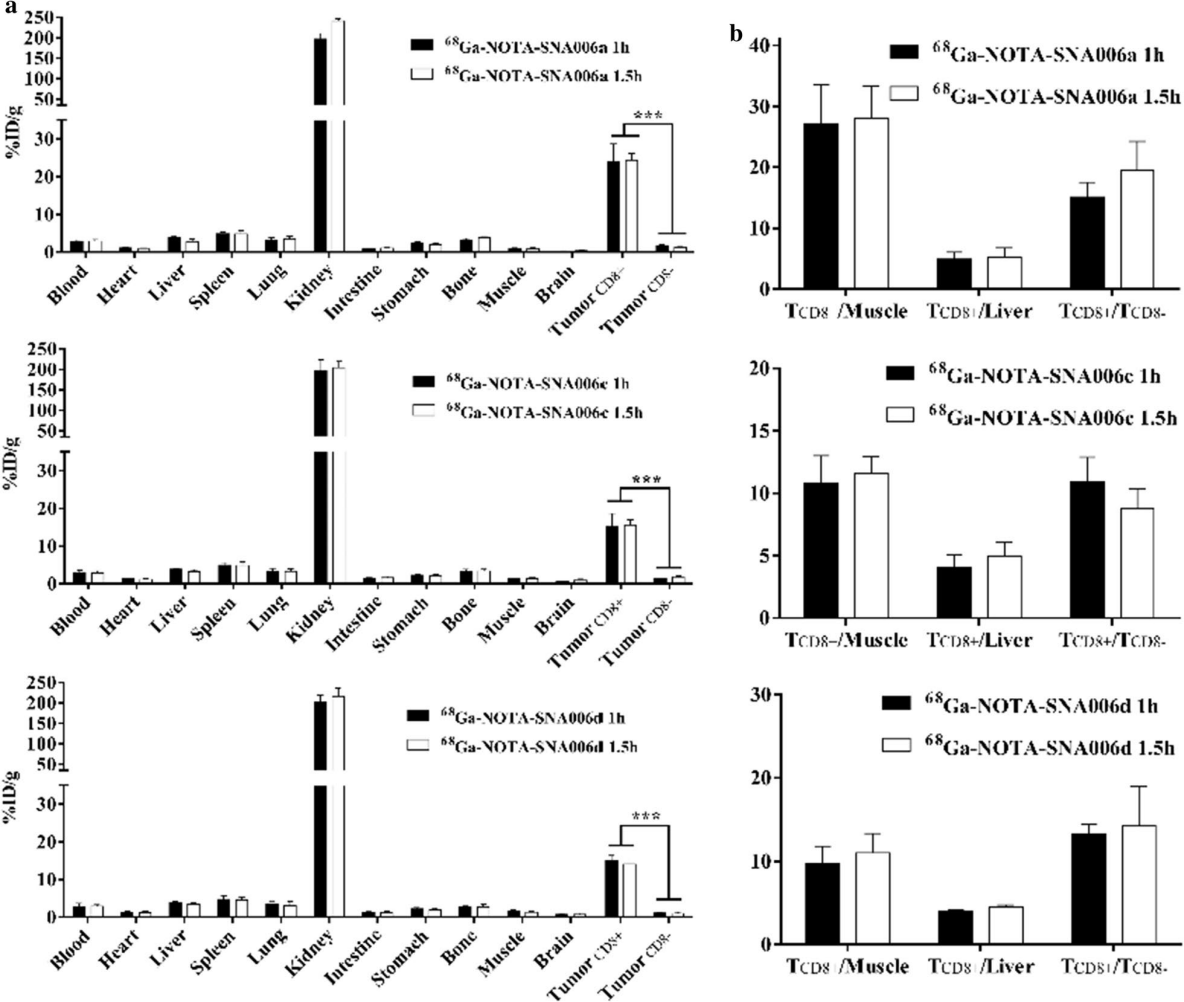
Biodistribution analysis (**a**) and tumor-to-background ratios (TBRs) (**b**) of ^68^Ga-NOTA-SNA006 at 1 and 1.5 h p.i. in both MC38-CD8 (CD8-positive) and MC38 (CD8-negative) tumor-bearing nude mice models (n = 4). ***p < 0.01

Biodistribution studies were also performed in mice bearing both CD8^+^ and CD8^−^ xenografts, and the results correlated well with the micro-PET/CT imaging experiments. As shown in Fig. 5, ^68^Ga-NOTA-SNA006a, c and d possessed similar in vivo pharmacokinetic characteristics. At 1 h p.i., the kidney showed the highest uptake (~ 200% ID/g) with low uptake in other normal organs such as spleen, liver, lung, and bones. (< 5% ID/g). The CD8^+^ tumor uptake was significantly higher than that in CD8^−^ tumor (24.08 ± 2.65% ID/g vs.1.59 ± 0.38% ID/g for ^68^Ga-NOTA-SNA006a, 15.34 ± 1.88% ID/g versus 1.40 ± 0.04% ID/g for ^68^Ga-NOTA-SNA006c, 15.06 ± 0.76% ID/g versus 1.14 ± 0.06% ID/g for ^68^Ga-NOTA-SNA006d at 1 h p.i., respectively). At 1.5 h p.i., the uptake in CD8^+^ tumors did not decrease significantly, accompanied by increase in the kidney uptake and decrease in most other normal organs.

Remarkable CD8^+^ tumor and low background uptake was observed for ^68^Ga-NOTA-SNA006a through the biodistribution studies. Notably, satisfactory TBRs in CD8^+^ tumor models were shown at all investigated time-points. For ^68^Ga-NOTA-SNA006a, the tumor-to-muscle, tumor-to-liver, CD8^+^ tumor-to-CD8^−^ tumor uptake ratio was 28.10 ± 3.68, 5.26 ± 0.86, and 19.58 ± 2.70, respectively (n = 4; Fig. 5b). Meanwhile, ^68^Ga-NOTA-SNA006c and d also possessed good CD8^+^ tumor uptake and TBRs.

### Human CD8 tracking

**Fig. 6:**
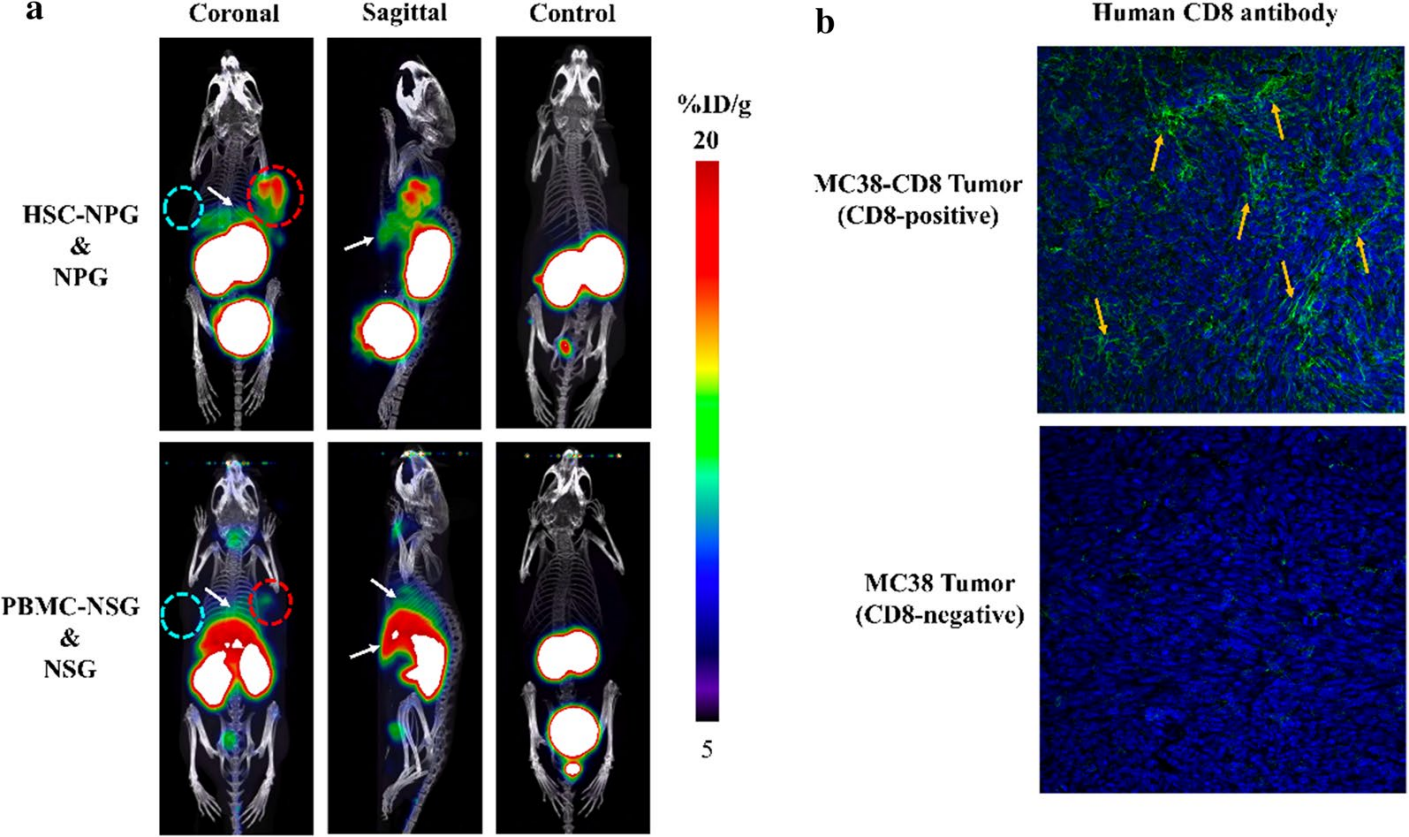
**a** The Micro-PET/CT imaging of ^68^Ga-NOTA-SNA006a in HSC-NPG and PBMC-NSG mice models bearing both types of tumors as well as control (NPG and NSG) mice at 1 h p.i., white arrows represent the uptake in normal tissue like lung, liver and spleen. **b** The human CD8 antigen immunohistochemical staining of tumors

The micro-PET/CT imaging in HSC-NPG and PBMC-NSG mouse models bearing CD8^+^ and CD8^−^ xenografts was conducted to further verify the ability of ^68^Ga-NOTA-SNA006a in tracking human CD8 antigen. As shown in Fig. 6a, ^68^Ga-NOTA-SNA006a was only concentrated in the kidney and bladder with clean background in the control NPG and NSG mice. In HSC-NPG and PBMC-NSG mouse models, significant uptake was observed in CD8^+^ tumors, lung, spleen and liver, and the CD8^+^ tumor uptake in HSC-NPG was higher than that in PBMC-NSG. Conversely, the uptake in liver, spleen and lung was higher in PBMC-NSG. There was also slight uptake in the thyroid in PBMC-NSG model. The expression of CD8 antigen in tumors, lung, liver and spleen of the PBMC-NSG mouse model was verified through immunohistochemistry and flow cytometry, and the results correlated well with the ^68^Ga-NOTA-SNA006a immunoPET imaging results in both types of models and tumors (Fig. 6b & Additional file 1: Fig. S6).

## Discussion

Immunotherapy has opened a new era of cancer treatment, and immune exhaustion or inhibition of lymphocytes especially CD8^+^ T cells in tumor microenvironment is considered as one of the most important reasons for inadequate immunotherapy effect [19]. A recent study showed that patients with kidney cancer whose tumor CD8^+^ T cell infiltration was less than 2.2% have a four-fold higher risk of disease progression after surgery [10]. In addition, quantitative immune scores based on tumor CD3^+^ and CD8^+^ lymphocytes infiltration predict more accurately than traditional TNM staging for the survival of colorectal cancer patients [20, 21]. Therefore, understanding the status of CD8^+^ T cells in vivo is critical for the prognosis of immunotherapy patients [4].

CDx relies on accurate and reliable diagnostic techniques to guide targeted therapy as well as immunotherapy. However, the most commonly used detection methods such as flow cytometry for blood samples and immunohistochemistry for tissue biopsy fail to provide comprehensive and dynamic information and have certain limitations. Moreover, it is challenging for ^18^F-FDG to distinguish between pseudoprogression and immune-related adverse events [22, 23]. ImmunoPET will provide great help for precisely stratifying patients into responders and nonresponders before treatment, and determining to continue or terminate therapy during the process or even predicting the patient outcome by tracking CD8^+^ T cells.

Antibody skeleton based immunoPET molecular probes targeting CD8^+^ T cells are in full bloom [24, 25], and have achieved encouraging progress. Among them, ^89^Zr-labeled minibody Df-IAB22M2C has passed preclinical evaluation and is currently undergoing phase II clinical trial [26]. Nanobodies overcome many deficiencies of other antibody derivatives, such as slow clearance, immunogenicity, poor penetrability, high price, long half-life nuclides demand due to their small size. Therefore, a series of ^68^Ga-labeled agents based on Nanobodies with high specific affinity to human CD8 antigen were designed and prepared. Among them, ^68^Ga-NOTA-SNA006a showed admirable ability in PET imaging of CD8 antigen in vivo, which held great potential for evaluation and monitoring of immunotherapy.

ELISA studies proved that the conjugation of chelator NOTA had no effect on the binding affinity, and confirmed the binding specificity of both SNA006 and NOTA-SNA006 to CD8 antigen. In the present case, the conjugation of NOTA-SNA006 was proceeded by a lysine-linked method. Given that, it is necessary to determine the impact of NOTA conjugation on SNA006 binding to CD8. The ELISA binding results for SNA006 Nanobodies with/without NOTA conjugation are shown in Additional file 1: Fig. S2. From these data, we concluded that NOTA conjugations exhibit similar binding affinity. These results support the availability of ^68^Ga-NOTA-SNA006 for CD8 detection. Although lysine-based conjugation processes yielded heterogeneous conjugates that were mixtures of species with different numbers of NOTA linked at different sites on the Nanobody surface. If further conjugation is proceeded by linkage of maleimide-NOTA to cysteine derived from reduction of interchain disulfides, the binding affinity might also be impacted by the reductants, such as dithiothreitol (DTT) or tris(2-carboxyethyl)phosphine (TCEP).

The in vitro binding assays not only fully demonstrated the binding specificity of SNA006 nanobodies to CD8 antigen, but also showed excellent *K*_*D*_ values. All of them specifically bind to CD8^+^ MC38-CD8 cells and rarely bind to CD8^−^ MC38 cells, which indicates a CD8-dependent manner. Although the values measured by different experimental methods varied, the binding capability of SNA006a to both CD8 antigen and CD8^+^ cells is strongest over the other two in vitro.

As shown in Figs. 4 and 5, the results of micro-PET imaging and biodistribution were basically consistent, and ^68^Ga-NOTA-SNA006a, c and d exhibited similar pharmacokinetic properties in vivo. Intense and increasing kidney retention was observed over time for all tracers, and they were excreted mainly through the urinary system due to the hexa-histidine tag components in their skeleton structures, which might require further optimization and improvement subsequently. The uptake of tracers in CD8^+^ tumors was significantly higher than that in CD8^−^ tumors, and CD8^+^ tumors can be clearly visualized through micro-PET imaging while CD8^−^ tumors cannot. The above results, together with ^18^F-FDG PET imaging and ^125^I-SNA006 SPECT imaging (Additional file 1: Fig. 5c), fully demonstrated their CD8-mediated targeting specificity in vivo. The rapid and persistent high retention in CD8^+^ tumors and gradually decreasing low uptake in CD8^−^ tumors and normal tissues such as liver and muscle over time lead to satisfactory TBR, which might also contribute to the detection of metastatic foci and the reduction of immune-related adverse events (irAEs). Among them, ^68^Ga-NOTA-SNA006a showed the most impressive characteristics in vivo with the best tumor imaging effect and highest tumor uptake and TBRs, which was consistent with binding results in vitro. Moreover, immunoPET imaging might be achieved at more early stage after drug administration, thus greatly reducing patient`s waiting time.

The micro-PET/CT imaging with ^68^Ga-NOTA-SNA006a in HSC-NPG and PBMC-NSG mice models was conducted to evaluate the human CD8 tracking ability. ^68^Ga-NOTA-SNA006a was mainly concentrated in the CD8^+^ tumor, liver, spleen, and lung (Fig. 6a) where human CD8^+^ T cells mainly aggregated or human CD8 antigen was overexpressed. As shown by the flow cytometry and immunohistochemical staining results (Fig. 6b & Additional file 1: Fig. S6), there was more human CD8 antigen expression in the PBMC-NSG mice, resulting in increased tracer uptake in the organs and tissues of the PBMC-NSG than that of the HSC-NPG mice. In parallel with this phenomenon, uptake in the CD8^+^ tumor of the HSC-NPG mice was higher compared to the corresponding uptake of the PBMC-NSG mice, presumably due to the relatively longer circulation time of the tracer in the former tumor models. The mild uptake in the thyroid of the PBMC-NSG mice was caused by the infiltration of the human CD8^+^ T cells.

## Concusions

A series of novel ^68^Ga-labeled Nanobody CDx tracers targeting human CD8 antigen were developed with high radiochemical purity. Among them, ^68^Ga-NOTA-SNA006a showed the highest affinity toward both CD8 protein and CD8^+^ cells in a CD8-dependent manner in vitro. It showed selective, rapid, high and sustained retention in the CD8^+^ tumor with low background in normal tissues (like the muscle, liver except for the kidney) and CD8^−^ tumor in vivo, resulting in impressive TBRs. Moreover, human CD8^+^ T cells could be precisely tracked through immunoPET imaging in PBMC-NSG and HSC-NPG mice models. The results fully demonstrated that ^68^Ga-NOTA-SNA006a possessed great potential for human CD8^+^ T cells detection, strategy formulation, efficacy evaluation and prognosis during immunotherapy in the context of precision medicine.

## Supplementary information

**Additional file 1.** Supplementary Figure 1-6.

## Data Availability

Not applicable.

## Abbreviations

immunoPET: Immuno-positron emission tomography
KD: The equilibrium dissociation constant
TBRs: Target-to-background ratios
CAR-T: Chimeric antigen receptor T cells
CDx: Companion diagnostics
p.i.: Post-injection
MS: Mass spectrometry
HPLC: High Performance Liquid Chromatography
PEI: Polyethylenimine
PBMC: Human peripheral blood mononuclear cell
HSC: Haemopoietic stem cell
SPR: Surface plasmon resonance
ROI: Regions of interest
