# Supplementary material for: ImmunoPET imaging of human CD8+ T cells with novel 68Ga-labeled nanobody companion diagnostic agents
Source: J Nanobiotechnology. 2021 Feb 9;19:42. doi: 10.1186/s12951-021-00785-9 (PMC7871532; doi:10.1186/s12951-021-00785-9)
Supplement: Supplementary file 1 — Additional file 1. Supplementary Figure 1-6. [file 12951_2021_785_MOESM1_ESM.docx]

**Supplementary Figure 1**


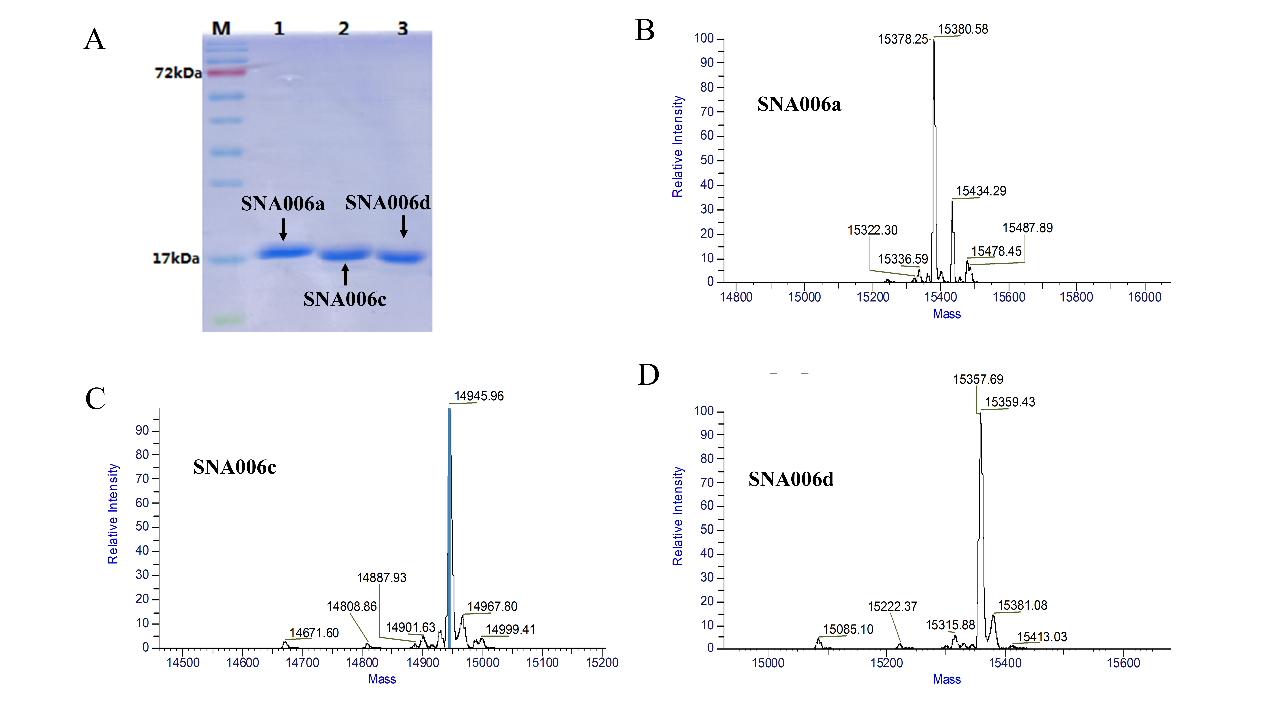


**Supplementary Figure 1** The SDS-PAGE (A) and ESI-Q-TOF-MS characterization of SNA006a (B), SNA006c (C) and SNA006d (D).

**Supplementary Figure 2**


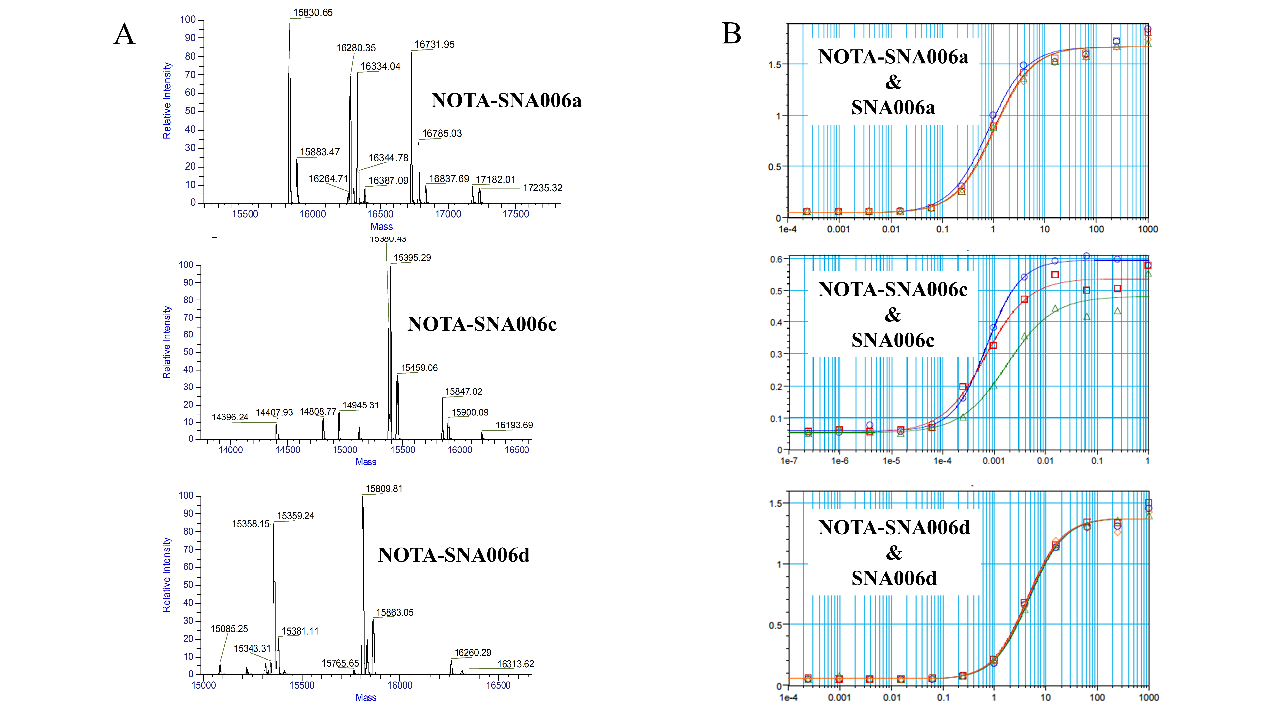


**Supplementary Figure 2** (A) The ESI-Q-TOF-MS characterization of NOTA-SNA006 (B) binding curves of SNA006 and NOTA-SNA006 to human CD8 determined by ELISA.

**Supplementary Figure 3**


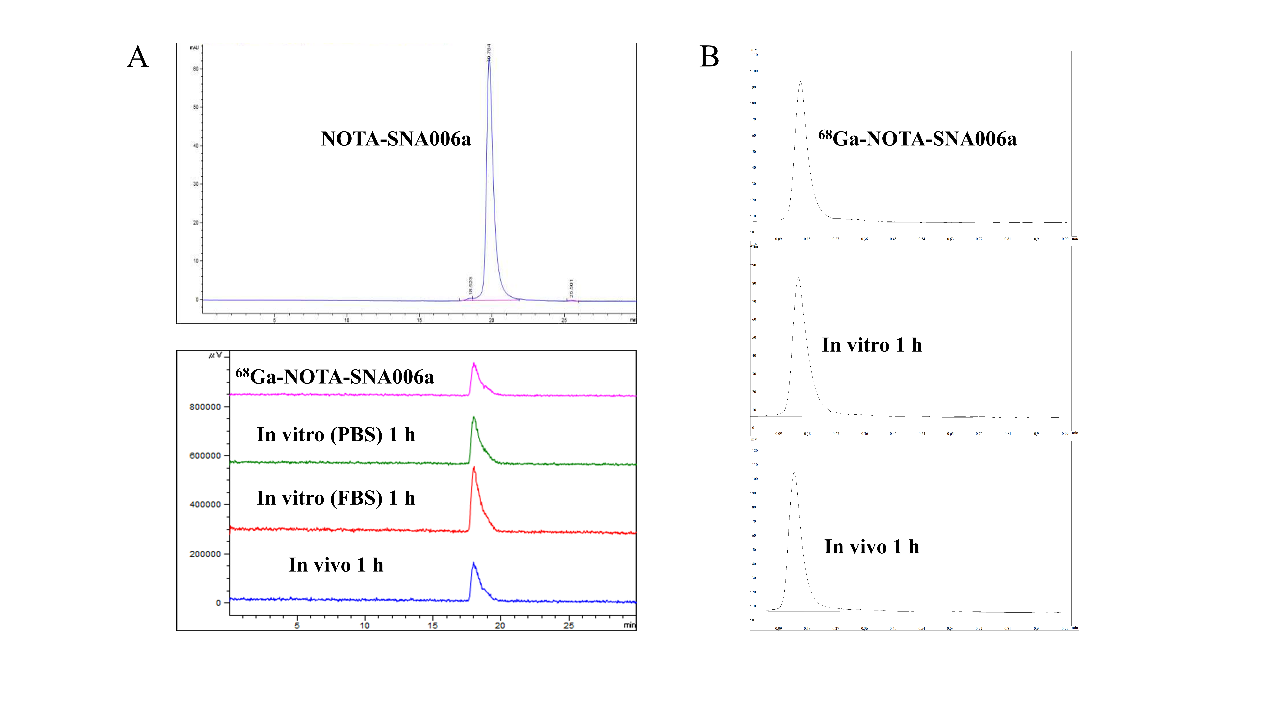


**Supplementary Figure 3** (A) The characteristics of NOTA-SNA006a and the stability of ^68^Ga-NOTA-SNA006a in vitro and in vivo (blood) determined HPLC: UV detector (320 nm), ^68^Ga-NOTA-SNA006a: radioactive detector. (B) The stability of ^68^Ga-NOTA-SNA006a in vitro and in vivo (blood) test by TLC.

**Supplementary Figure 4**


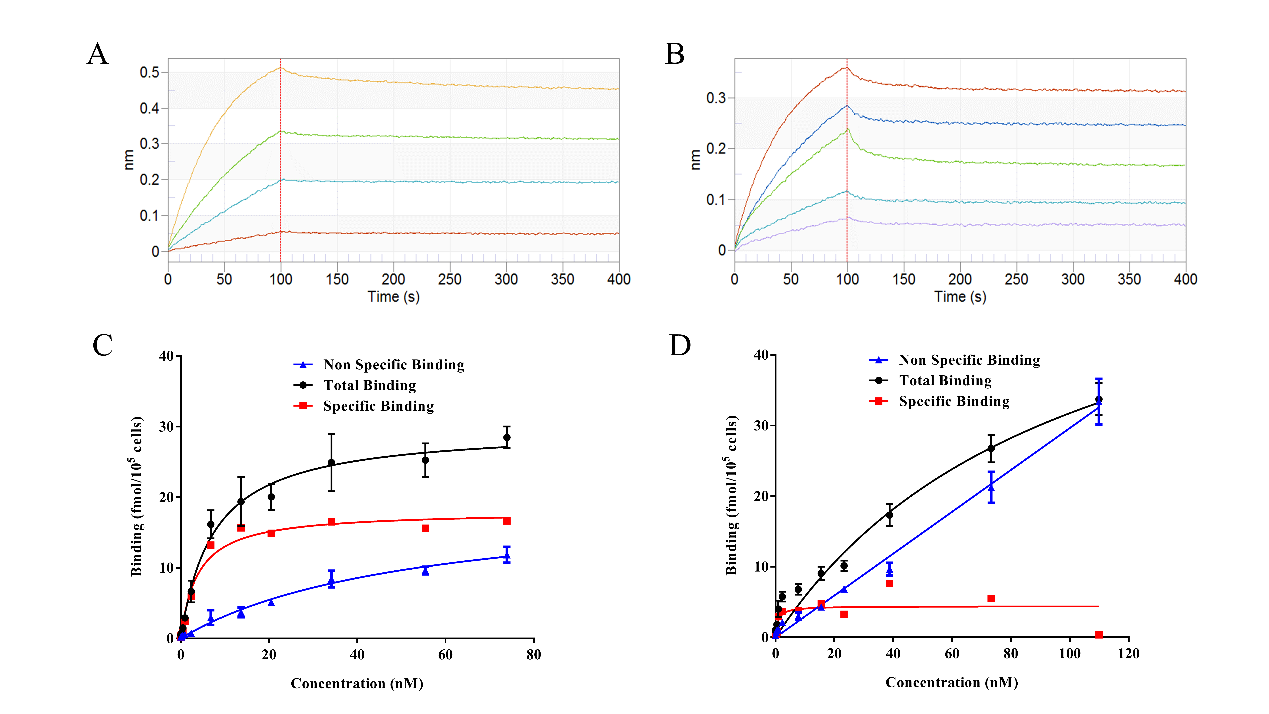


**Supplementary Figure 4** Affinity/kinetics SPR study of SNA006c (A) and SNA006d (B) interacting with immobilized recombinant human CD8 protein. The saturation binding curve of ^125^I-SNA006c (C) and ^125^I-SNA006c (D) to MC38-CD8 cells at 4 h.

**Supplementary Figure 5**


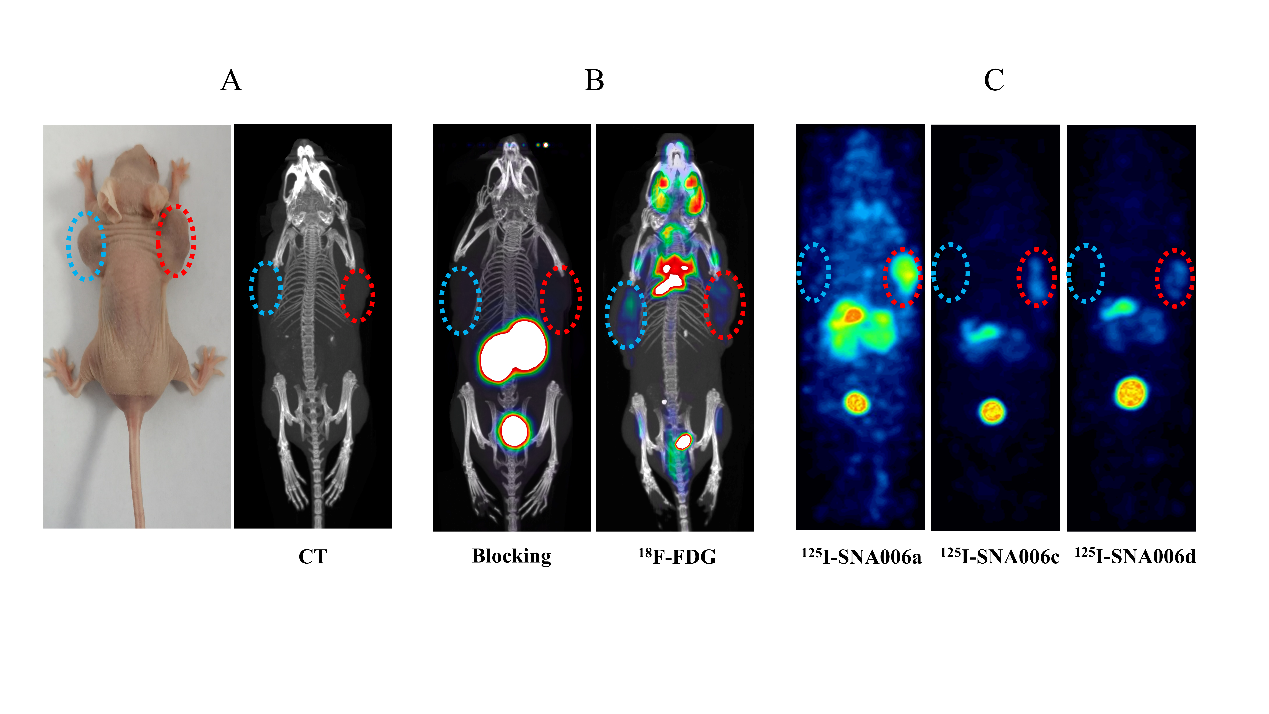


**Supplementary Figure 5** (A) BALB/c nude mouse model bearing both CD8^+^ MC38-CD8 (right, red circle) and CD8^-^ MC38 (blue circle) as well as CT image. (B) ^18^F-FDG PET/CT imaging and the blocking imaging at 1 h post co-injection of 3.7 MBq of ^68^Ga-NOTA-SNA006a and SNA006a in tumor-bearing BALB/c nude mice models. (C) ^125^I-SNA006 SPECT imaging in the above mice models at 1 h.

**Supplementary Figure 6**


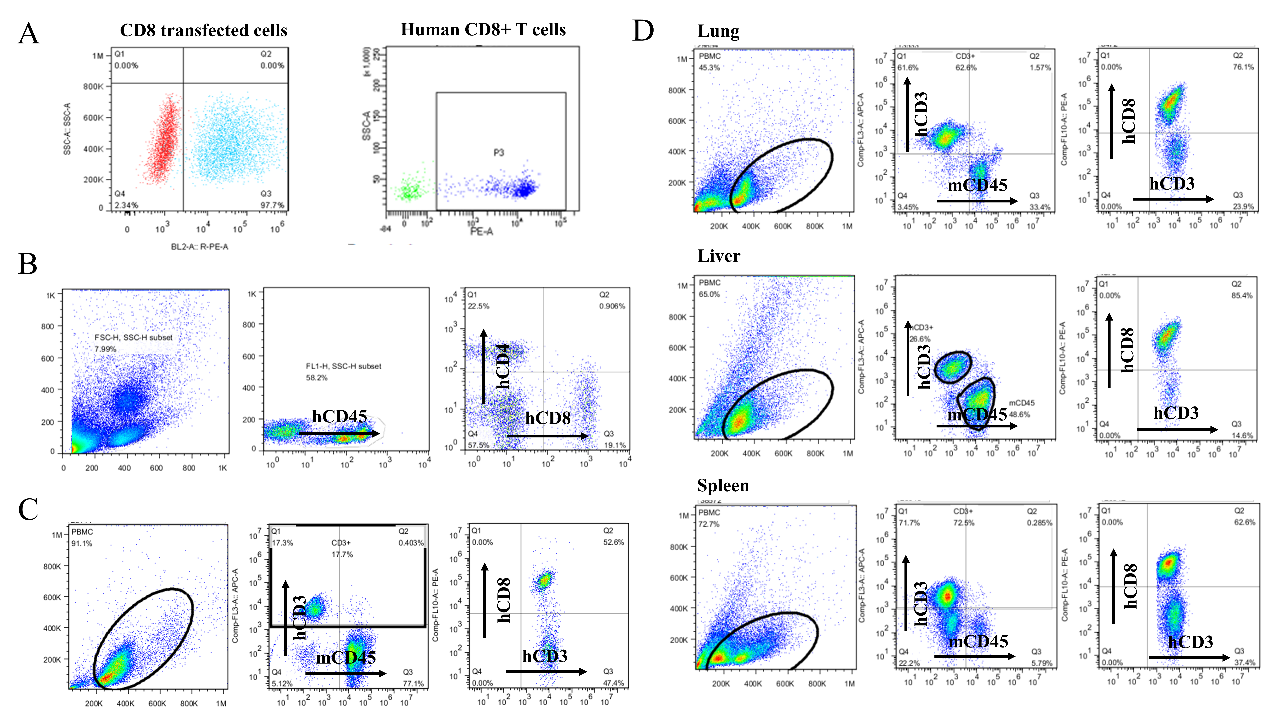


**Supplementary Figure 6** The expression of human CD8 antigen in MC38-CD8 (CD8-positive) and human CD8^+^ T cells (A), HSC-NPG mouse model (B), PBMC-NSG mouse model (C) and normal organs like lung, liver and spleen of PBMC-NSG mouse model (D) by flow cytometry.
